# Supplementary material for: Intermolecular Non-Covalent Carbon-Bonding Interactions with Methyl Groups: A CSD, PDB and DFT Study
Source: Molecules. 2019 Sep 16;24(18):3370. doi: 10.3390/molecules24183370 (PMC6767229; doi:10.3390/molecules24183370)
Supplement: Supplementary file 1 [file molecules-24-03370-s001.pdf]

molecules

Electronic Supporting Information
accompanying:

Intermolecular non-covalent Carbon-bonding interactions with methyl groups: a CSD, PDB and DFT study

Tiddo J. Mooibroek

van 't Hoff Institute for Molecular Sciences, Universiteit van Amsterdam, Science Park 904, 1098 XH Amsterdam, The Netherlands; E-mail: t.j.mooibroek@uva.nl

Contents

Table S1. Numerical overview of datasets retrieved from the CSD and the PDB……………………………………….2

Table S2. Numerical overview data characterized by $\alpha \geq 160°$………………………………………………………………….3

Figure S1. ***P*** (α) directionality plots for X–CH_3···ElR pairs in the CSD and the PDB………………………………………4

Figure S2. Cumulative *N*(***d'***) plots of the regular *N*(***d'***) plots shown in Figure 3…………………………………………..5

Figure S3. *N*(***d'***) plots for datasets not features in the main text of the paper……………………………………………….6

Figure S4. Perspective views and 'atoms-in-molecules' analyses of adducts **1**-**21**…………………………………….7

Figure S5. Perspective views and 'atoms-in-molecules' analyses of adducts with benzene………………………….8

References………8

Table S1. Numerical overview of datasets retrieved from the CSD and the PDB. See Figure S1 and Figure 2 for ***P*** (α) directionality plots. For data of refined datasets see Table S2 ($\alpha \geq 160°$ and van der Waals overlap). 'n.a.' stands for 'not assessed'. The data for quaternary (cationic) N-atoms is not used in the paper for ***P***(α) plot but they were used in *N*(***d'***) plots.

Interacting group	Methyl	CSD data		PDB data	
		CIFs	Hits	PDBs	Hits
Water (O)	$H_3C–C$	14,163	46,000	11,426	29,508
	$H_3C–N$	4,696	18,170	6,049	17,101
	$H_3C–O$	3,026	7,190	4,999	11,392
	$H_3C–P$	108	520	48	86
	$H_3C–S$	528	1,546	8,718	30,725
Amide (O)	$H_3C–C$	18,782	53,473	9,809	22,538
	$H_3C–N$	3,316	7,663	6,100	10,855
	$H_3C–O$	5,075	9,158	8,361	11,064
	$H_3C–P$	37	81	34	48
	$H_3C–S$	860	2,175	8,702	11,215
Carboxyl (any O)[a]	$H_3C–C$	60,406	294,952	16,884	27,698
	$H_3C–N$	7,799	31,260	2,669	4,943
	$H_3C–O$	24,931	96,082	2,008	3,314
	$H_3C–P$	1,454	1,454	50	83
	$H_3C–S$	1,420	5,226	3,441	5,451
Sulphur (CS)	$H_3C–C$	17,508	54,927	9,751	13,792
	$H_3C–N$	3,027	7,431	1,025	1,296
	$H_3C–O$	3,520	7,086	1,528	1,772
	$H_3C–P$	153	480	3	3
	$H_3C–S$	3,055	9,200	500	562
Aryl (centroid)	$H_3C–C$	242,033	888,217	2,079	3,261
	$H_3C–N$	42,811	119,381	1,493	3,280
	$H_3C–O$	59,217	146,996	2,681	2,811
	$H_3C–P$	5,348	20,706	45	47
	$H_3C–S$	7,013	18,252	1,999	2,757
$C_3N^+–CH_3$	Amide	479	3,483	n.a.	
	Water	130	446	n.a.	
	Carboxyl	426	3,344	n.a.	
	CS	149	871	n.a.	
	Aryl	1,456	5,265	n.a.	

Table S2. Numerical overview of the amount of data in each dataset from Table S1 characterized by $\alpha \geq 160°$ ($N^{\alpha \geq 160°}$), together with the amount of data within that dataset where van der Waals radii overlap ($N^{\Sigma vdW}$). 'n.a.' stands for 'not assessed'. Datasets with $N^{\alpha \geq 160°}$ larger than 100 were inspected further by means of the $N(\boldsymbol{d'})$ plots shown in Figures S2–S3. As a guide to the eye, datasets with less than 100 hits are in grey, those between 100 and 500 hits are in blue and those above 500 are in regular black.

		OH_2 (water)		O = C (amide)		O_2C (carboxyl)		SC (Thio)		Centroid (aryl)	
		**CSD**	**PDB**	**CSD**	**PDB**	**CSD**	**PDB**	**CSD**	**PDB**	**CSD**	**PDB**
**C–CH$_3$**	$N^{\alpha \geq 160°}$	2,376	1,622	2,599	1,178	16,857	2,288	3,420	835	70,690	107
	$N^{\Sigma vdW}$	72 (3.0%)	443 (27%)	104 (4.0%)	210 (18%)	574 (3.4%)	193 (8.4%)	133 (3.9%)	83 (9.9%)	402 (0.6%)	4 (3.7%)
**N–CH$_3$**	$N^{\alpha \geq 160°}$	1,089	1,186	504	650	2,062	286	504	89	10,585	93
	$N^{\Sigma vdW}$	162 (15%)	398 (34%)	135 (27%)	199 (31%)	378 (18%)	70 (25%)	59 (12%)	-	134 (1.3%)	-
**O–CH$_3$**	$N^{\alpha \geq 160°}$	452	757	640	483	6,927	168	578	114	11,009	77
	$N^{\Sigma vdW}$	69 (15%)	285 (38%)	181 (28%)	152 (32%)	1,409 (20%)	47 (28%)	74 (13%)	26 (23%)	289 (2.6%)	-
**P–CH$_3$**	$N^{\alpha \geq 160°}$	15	8	2	-	79	6	31	-	1,662	2
	$N^{\Sigma vdW}$	-	-	-	-	-	-	-	-	6 (0.4%)	-
**S–CH$_3$**	$N^{\alpha \geq 160°}$	89	1,368	78	579	315	390	553	61	1,417	73
	$N^{\Sigma vdW}$	-	464 (34%)	-	118 (20%)	41 (13%)	102 (26%)	41 (7.4%)	-	39 (2.8%)	-
**C$_3$N$^+$–CH$_3$**	$N^{\alpha \geq 160°}$	290	n.a.	42	n.a.	274	n.a.	48	n.a.	527	n.a.
	$N^{\Sigma vdW}$	78 (27%)		-		72 (26%)		-		12 (2.3%)	

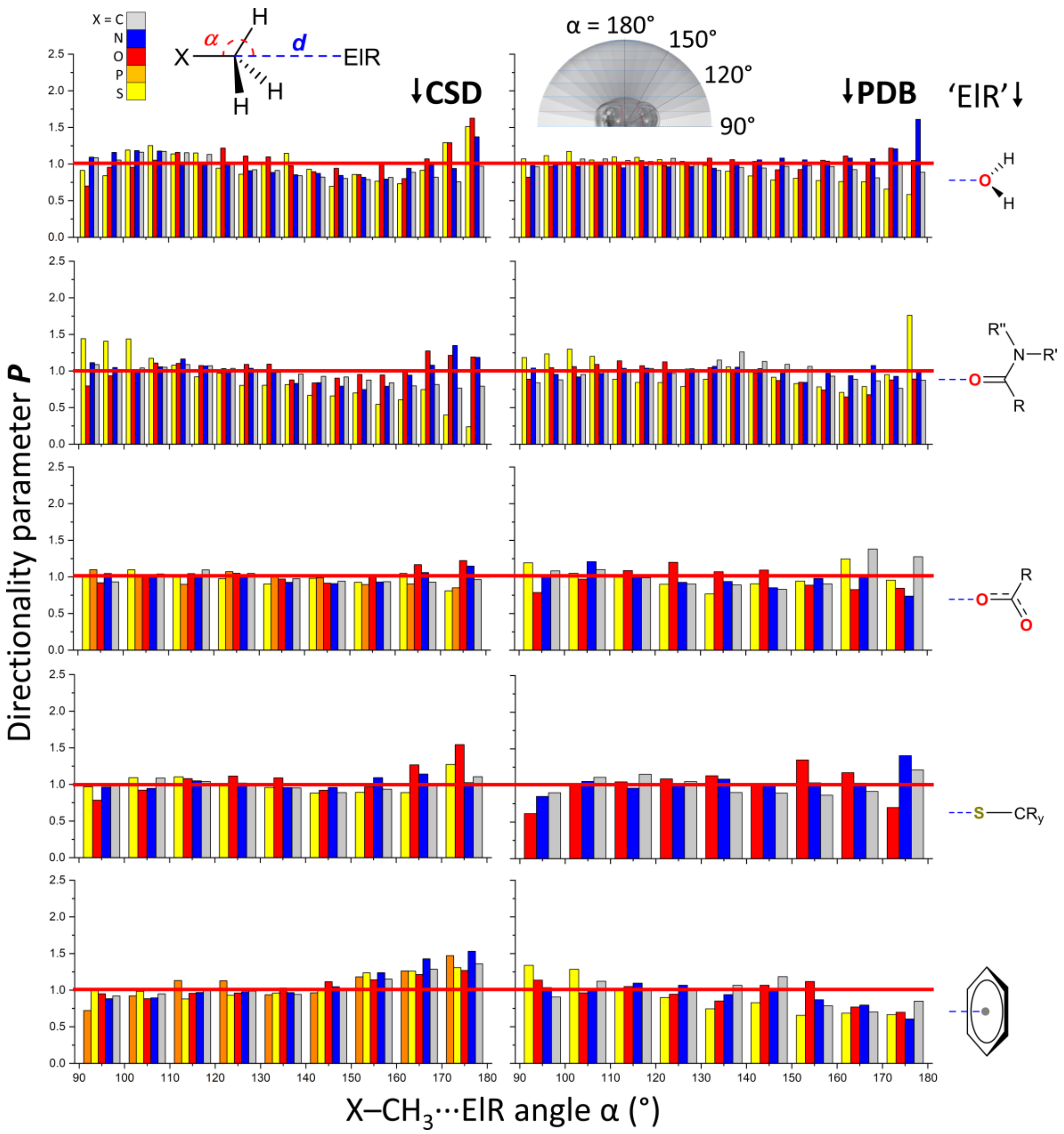

Figure S1. ***P*** (α) directionality plots for the data retrieved from the CSD (left) and the PDB (right) using the general query shown in the top-left inset figure for X–CH_3···ElR pairs. X can be C, N, O, P, or S and 'ElR' can be a water, amide or carboxyl O-atom, an R_yCS S-atom (y = 2 or 3, R = any non-metal) or the centroid of an aryl ring, as is indicated in the right-hand side of the figure. The insert figure in the top right is intended as a guide to the eye to interpret the spatial location of data with a certain value of α. Due to the amount of data per dataset (see Table S1 for numerical overview), the top four ***P*** (α) plots are given at a 5° resolution for α and the bottom six at a 10° resolution. **NB:** Interestingly, in the ***P***(α) plots for X = S and ElR = water, ***P*** is above unity at α = 160°–180° for the CSD data, while ***P*** < 1 for the PDB data in this same region. In both databases, the ***P***-values are ≥ 1 around α = 105°. This indicates that the H-bonding geometry (α ≈ 105°) is somewhat directional on both databases but that the carbon bonding geometry is more directional only in the CSD. However, the dataset retrieved from the CSD is much smaller (1,546 hits) than the dataset from the PDB (30,725 hits) and the observed feature α = 160°–180° in the CSD might well be an artefact. Similarly, the ***P***(α) plots with X = S and an amide O-atom reveal that ***P*** ≥ 1 at α = 90°–105°, again congruent with a hydrogen bonding geometry. ***P*** ≥ 1 also at α = 170°–180, but only for the PDB data. As this dataset is more voluminous (*N* = 11,215 *vs* 2,175 in the CSD), this implies that a carbon bonding geometry is more directional (at least in protein structures). A possible reason for this discrepancy might be that many cysteine and methionine residues are involved in metal coordination or directly methylated thus polarizing the S–C bond. For example, iron-sulphur clusters are held in place by cysteine–Fe

coordination bonds[1] and methylated methionine residues are a (crystallographically known) intermediate in methyl transferases.[2].

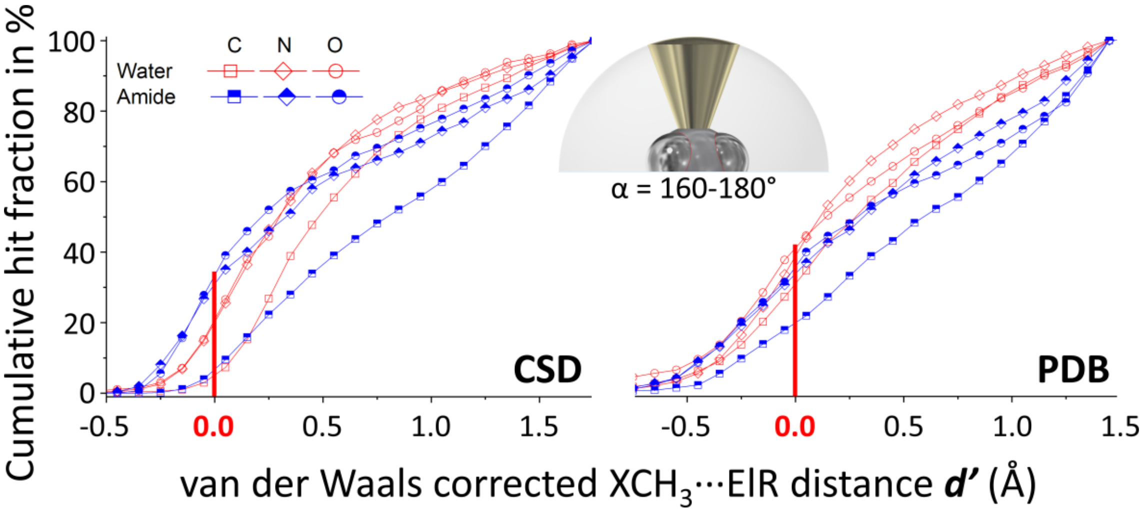

Figure S2. Cumulative hit fraction (in %) as a function of the van der Waals corrected $XCH_3\cdots ElR$ distance ***d'*** (in Å) for several datasets from Table S2 ($\alpha \geq 160°$, as illustrated by the inset figure). The interacting pairs involve water-O (red, empty) or amide-O (blue, half-filled) with X = C (squares), N (diamonds), or O (circles). See Figure 3 for the same data plotted as a regular hit fraction.

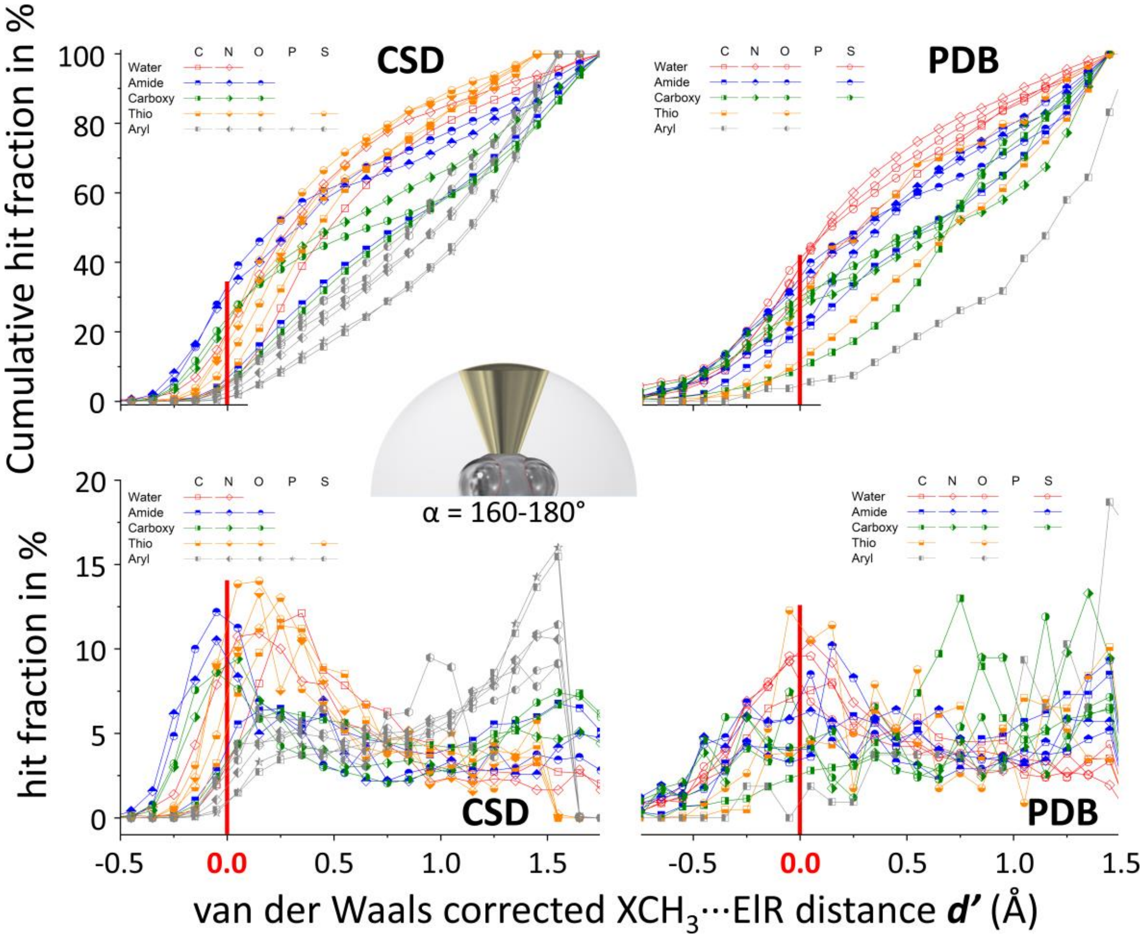

Figure S3. Cumulative (top) and regular (bottom) hit fraction (in %) as a function of the van der Waals corrected $XCH_3\cdots ElR$ distance ***d'*** (in Å) for all the datasets from Table S2 ($\alpha \geq 160°$, illustrated with the inset figure) that contain ≥ 500 data points. The inset legends show the nature of X (horizontally) and ElR (vertically). **NB:** In addition to the trends observed and discussed in the main text according to

Figure 3, It is interesting to note that in all cases, for X = C the plot is shifted most to longer ***d'*** and has the least van der Waals overlap. Moreover, carboxy O-atoms are distributed about the same as amide O-atoms and thio S-atoms about the same as water. Aryls are always randomly distributed with the least amount of van der Waals overlap.

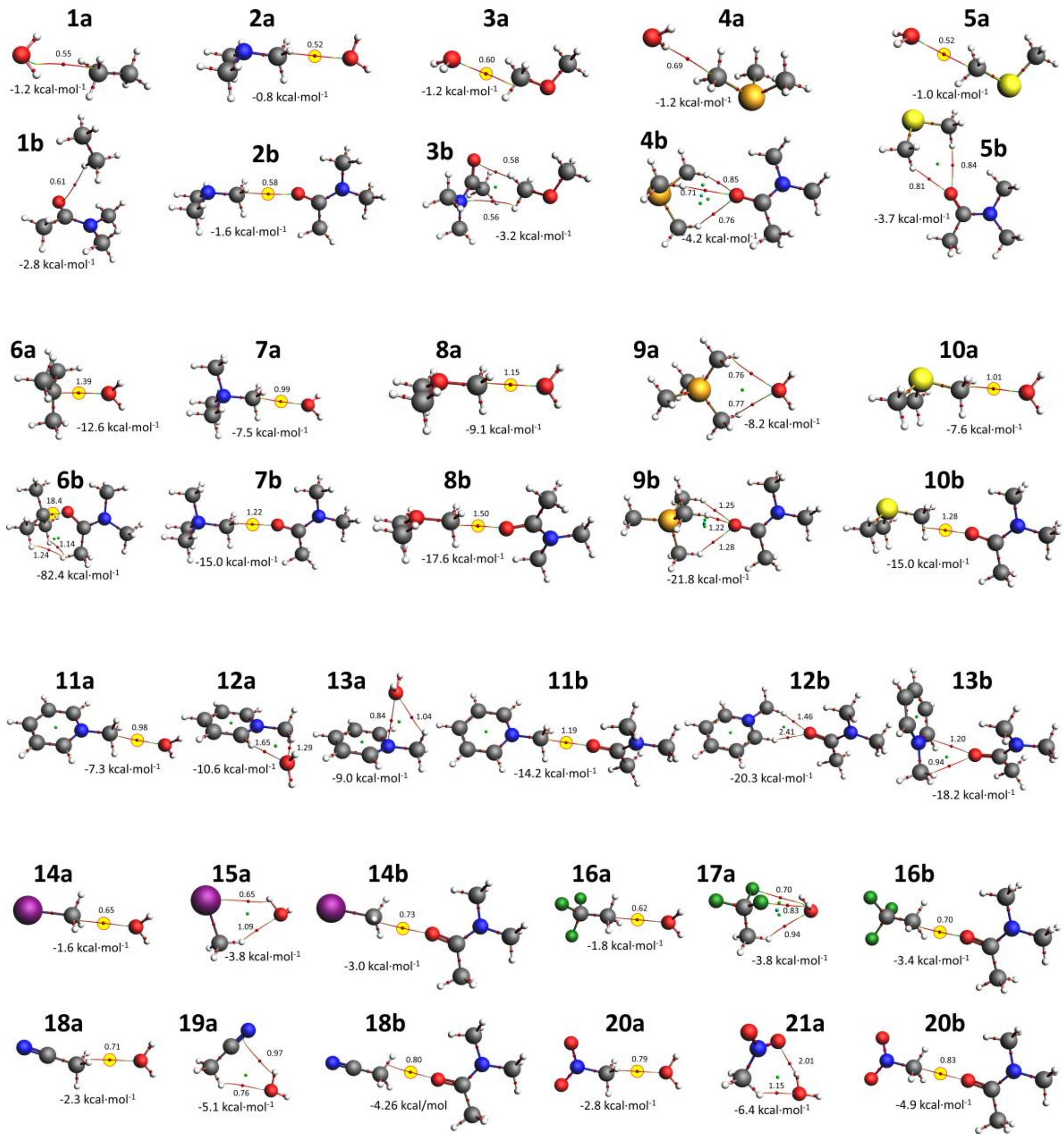

Figure S4. Ball and stick representations of perspective views of all molecular adducts listed in Table 1 that were optimized by DFT (B3LYP-D3/def2-TZVP). The thin lines are bond paths (bp's) and the small red spheres are bond critical points (bcp's) obtained from an 'atoms-in-molecules' analysis. The bond density (ρ) is in arbitrary units $\cdot 10^2$ and bcp's indicative of non-covalent carbon bonding have been highlighted in yellow.

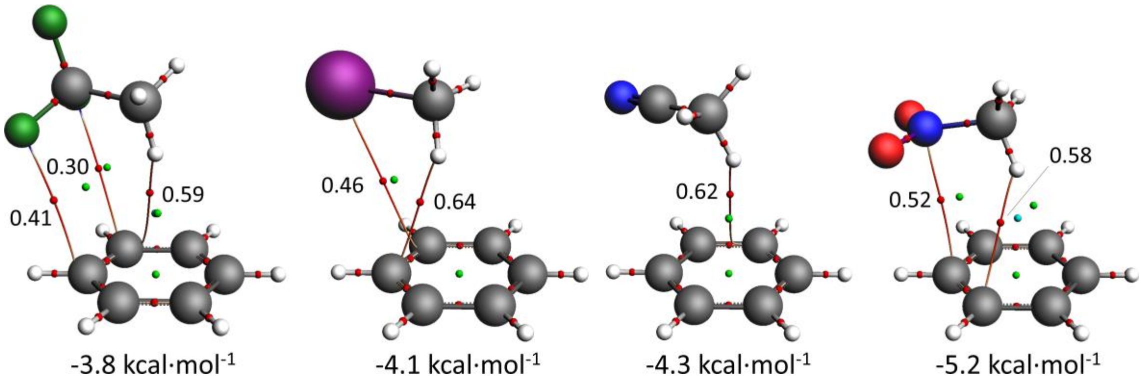

Figure S5. Ball and stick representations of molecular adducts selected from Table 1 that were optimized by DFT (B3LYP-D3/def2-TZVP). The thin lines are bond paths (bp's) and the small red spheres are bond critical points (bcp's) obtained from an 'atoms-in-molecules' analysis. The bond density (ρ) is in arbitrary units $\cdot 10^2$ and bcp's indicative of non-covalent carbon bonding have been highlighted in yellow.

References

1. H. Beinert, R. H. Holm, E. Munck, *Science* **1997**, *277*, 653–659
2. D. C. Johnson, D. R. Dean, A. D. Smith, *et al.*, *Annu. Rev. Biochem.* **2005**, *74*, 247–281.
3 H. L. Schubert, R. M. Blumenthal, X. D. Cheng, *Trends Biochem. Sci.* **2003**, *28*, 329–335.

© 2019 by the author. Licensee MDPI, Basel, Switzerland. This article is an open access article distributed under the terms and conditions of the Creative Commons Attribution (CC BY) license (http://creativecommons.org/licenses/by/4.0/).
